# Identifying specific needs in adult cystic fibrosis patients: a pilot study using a custom questionnaire

**DOI:** 10.1186/s12890-021-01613-4

**Published:** 2021-08-18

**Authors:** Sandra Dury, Jeanne-Marie Perotin, Bruno Ravoninjatovo, Catherine Llerena, Julien Ancel, Pauline Mulette, Muriel Griffon, Sophie Carré, Amélie Perrin, François Lebargy, Gaëtan Deslée, Claire Launois

**Affiliations:** 1 Department of Respiratory Diseases, Reims University Hospital, Maison Blanche University Hospital, 45, rue de Cognacq-Jay, 51 092 Reims cedex, France; 2 EA 4683 Medical and Pharmacological, University of Reims, Reims, France; 3 INSERM UMRS 1250, Reims University Hospital, Reims, France; 4 Department of Pediatrics, Grenoble University Hospital, Grenoble, France; 5 Department of Pediatrics, Nantes University Hospital, Nantes, France

**Keywords:** Cystic fibrosis, Needs, Questionnaire, Quality of life, Fatigue

## Abstract

**Background:**

Adult patients with cystic fibrosis (CF) experience daily physical symptoms and disabilities that can be challenging to address for health care teams.

**Methods:**

We sought to identify the most frequent topics that CF adults need to discuss with health care teams using a custom questionnaire including 62 items.

**Results:**

Fifty patients were included, 70% men, mean age 27.6 years, with a mean body mass index of 21.8 kg/m^2^. Mean FEV_1_% was 64% of predicted value. Forty-two percent of patients selected at least one topic. The most frequently selected topics were fatigue (20%), professional or scholar worries (18%), procreation (16%), physical activities (16%) and evolution of CF disease (16%). Women were more frequently concerned about fatigue, procreation and profession/school.

**Conclusions:**

Using a custom questionnaire, we identified that CF adults express various unmet needs that extend beyond usual respiratory and nutritional concerns or treatment adherence. The interest of this questionnaire by health care team for improving therapeutic management of CF patients remains to be validated.

*Trial registration*: The study was registered on ClinicalTrials.gov (NCT02924818) on 5th October 2016.

## Background

Cystic fibrosis (CF) is the most common life-threatening genetic disease in the USA and Caucasian populations. CF prognosis depends primarily on chronic respiratory failure and malnutrition, which are the focus of attention during follow-up visits [1, 2]. Thanks to advances in pharmacological treatments and improvements in patients care, CF patients’ survival dramatically increased. The median predicted survival for USA CF patients born in 2019 was 48.4 years [3].

Consequently, health care teams will need to serve a growing population of adults living with CF as a chronic illness. CF adults experience daily physical symptoms (cough, shortness of breath, lack of energy, nasal discharge) that significantly impair their functional status and quality of life [4, 5]. Patients also suffer from psychological symptoms including anxiety, irritability, sleeping troubles and sadness [4].

A few studies analysed expectations and unmet needs of CF adult patients. Sawicki et al*.* [6] reported that one-third of 18–24 years old CF patients required information about “ways to deal with decreased energy”, “new CF therapies”, and “ways to deal with the unpredictability of the future”. In a recent survey, CF patients reported a high level of confidence in their abilities in nutritional care and the role of enzymes in CF therapy but a low confidence in mental health and ability to have children [7]. Interestingly, CF patients have significant unmeet existential needs that are most prevalent in patients with higher symptom burden [8]. As part of a multidisciplinary evaluation, health care teams involved in CF management should not exclusively consider usual indicators of disease severity (e.g., lung function, body mass index, exercise performance and pulmonary exacerbations) to evaluate CF patients [9].

The objective of our study was to identify the most frequent topics that CF adults need to discuss with health care team during regular visits. We used an individual educational custom questionnaire available for French health care teams since 2013 [10]. This tool consists in a list of predefined topics covering the main domains of social, professional, familial and sexual life, psychological feeling, symptoms related to CF, daily life organisation, hobbies, treatments and life project.

## Methods

### Study protocol

CF adult patients were prospectively recruited from the University Hospital of Reims—Department of Respiratory Diseases between November 2016 and December 2019. Patients were included in the RINNOPARI study (Recherche et INNOvation en PAthologie Respiratoire Inflammatoire), an observational cohort of inflammatory chronic lung diseases. The study was approved by the Ethics Committee of Dijon EST I on 31rd October 2016 (N°2016-A00242-49) and by the French National Agency for Medicines and Health Products (ANSM) on 25th April 2016. The protocol was registered on ClinicalTrials.gov (NCT02924818) on 5th October 2016. Each patient signed a written informed consent form. All methods including recruitment, data collection and analysis have been performed in accordance with the declaration of Helsinki.

All participants were recruited for a regular CF care visit. Patients were included if they were at least 18 years of age. Exclusion criteria were previous or planned lung transplantation and patients requiring an urgent visit. Anonymised collected data included demography, clinical characteristics, pulmonary function tests results and sputum microbiological data. Quality of life was evaluated using the St George’s Respiratory Questionnaire (SGRQ) assessing symptoms and impact on daily activities (a maximum of 100 score indicates maximum impairment of quality of life) [11]. Chronic infection by *Pseudomonas aeruginosa,* and by extension chronic infection by *Staphylococcus aureus*, *Stenotrophomonas maltophilia* or *Achromobacter xylosoxidans* was defined according to Leeds criteria [12].

### Unmet needs questionnaire

“Des mots pour le dire” (literally “Words to say it”) is an individual educational questionnaire available since 2013 in French language. This questionnaire was developed by the workshop GETHEM (Groupe Education THErapeutique et Mucoviscidose) in order to improve medical visits by identifying CF patient needs [10]. This structured CF-specific questionnaire focuses on symptoms and feelings than are infrequently assessed during usual medical visit, including treatment burden, impact of illness on daily life and personal projects. The questionnaire consists of a list of 62 predefined topics divided in nine domains: social and familial life, professional life, sexual life, psychological feeling, symptoms related to CF, daily life, hobbies, treatments and projects. It also includes an open-ended question regarding items judged as irrelevant and too frequently raised during visits.

Included patients were asked to complete the questionnaire just before a visit by selecting the topics they need to discuss during the medical visit and circle the three most important topics to address.

### Statistical analysis

Data were described as numbers (percentages) or mean ± standard deviation or median [interquantile range] depending on distribution. Differences in clinical characteristics were assessed using chi-square tests or Fisher’s exact tests, as appropriate, for qualitative variables, and Student t-tests or Mann–Whitney U-tests for quantitative variables. A *p* value < 0.05 was considered statistically significant. Results were analysed with SPSSv26.

## Results

Fifty-one consecutive CF patients were included in the study. One patient was excluded because of previous lung transplantation. Fifty patients were analysed. No patient declined the study.

### Patients characteristics

Demographic and clinical characteristics of patients are detailed in Table 1. Briefly, patients were mostly men (70%), mean aged 27.6 ± 8.7 years with a mean body mass index of 21.8 ± 3.3 kg/m^2^. Two third were employed and or student, 67.3% lived as a couple. A medical history of depression was present in 15.7% of CF-patients.

**Table 1:** Demographic and Clinical Characteristics

Variables	
Male	35 (70%)
Age (years)	27.6 ± 8.7
BMI (kg/m^2^)	21.8 ± 3.3
Smoker	5 (9.8%)
Employment status	
Student	14 (27.4%)
Working	25 (49.0%)
None	16 (32.0%)
Family status	
Maried/cohabiting	33 (67.3%)
Living with their parents	16 (32.6%)
Comorbid illnesses	
Pancreatic insufficiency	40 (80.0%)
Diabetes	17 (34.0%)
Depression	8 (15.7%)
Symptoms	
Chronic cough	40 (80.0%)
Chronic expectoration	45 (90.0%)
Dyspnea	26 (52.0%)
Exacerbation in the last year	35 (70.0%)
Number of episodes per patient	2.3 ± 1.4
SGRQ	
Total	25.5 ± 17.3
Impact	19.7 ± 15.8
Activity	33.8 ± 21.0
Symptoms	45.0 ± 20.6

Data are expressed as frequency (percentage), mean ± standard deviation or median (Interquartile range)

*BMI* body mass index, *SGRQ* St George’s Respiratory Questionnaire

The mean SGRQ score was 25.5 ± 17.3 with a predominant impairment in the symptoms domain (45 ± 20.6). Seventy percent of the patients presented at least one respiratory exacerbation in the previous year, with a mean of 2.3 ± 1.4 events/patient/year.

Spirometric and microbiological data are detailed in Table 2. Mean FEV_1_% was 64 ± 30% of predicted value. Chronic infection by *Staphylococcus aureus* and *Pseudomonas aeruginosa* were present in 74% and 40% of the patients, respectively.

**Table 2:** Lung function and microbiology data

Variables	
Spirometry	
FEV_1_, % predicted	64 ± 30
6-Min walk distance	
Distance, m	562 ± 86
Minimal saturation, %	92 ± 7
Blood gases on air	
PaO_2_, mmHg	87 ± 20
PaCO_2_, mmHg	39 ± 5
Chronic colonisation	
*Pseudomonas aeruginosa*	20 (40.0%)
*Staphylococcus aureus*	37 (74.0%)
*Achromobacter xylosoxidans*	2 (4.0%)
*Stenotrophomonas maltophilia*	1 (2.0%)
Microbiological data at inclusion (n = 42, sputum)	
*Pseudomonas aeruginosa*	17 (40.5%)
*Staphylococcus aureus*	32 (76.2%)
*Achromobacter xylosoxidans*	1 (2.4%)

Data are expressed as frequency (percentage) or mean ± standard deviation

*FEV*_*1*_ forced expiratory volume at first second

### Unmet needs

Twenty-four patients (48%) did not express any unmet needs (Table 3). There was no significant difference in baseline characteristics between patients selecting at least one unmet need and those who did not. Patients selected a median of 1 [4.75] topics, twenty-six patients (52%) indicated at least one topic; five patients selected more than 10 topics. The most frequently selected topics were “fatigue” (20%), “study or job” (18%), “ability to conceive a child” (16%), “physical activity and sports” (16%) and “disease evolution” (16%). A lower number of patients reported “cough” (12%), “medical advances” (12%), “mood” (12%), several aspects of daily life organization (“leisure activity” (10%), “trip abroad” (8%)) and “personal projects” (8%) or “professional projects” (10%).

**Table 3:** Unmet needs

Domains	Requested domains		Prime domains	
Social and familial life				
Family	2	4.0%	0	0.0%
Couple	3	6.0%	0	0.0%
Children	2	4.0%	0	0.0%
Friends	3	6.0%	0	0.0%
Romantic encounter	1	2.0%	0	0.0%
Risky driving	2	4.0%	0	0.0%
To have a child	8	16.0%	4	8.0%
To discuss of specific illness	1	2.0%	0	0.0%
Professional life				
Study/job	9	18.0%	2	4.0%
Internship abroad	1	2.0%	1	2.0%
Sick leave	2	4.0%	1	2.0%
Social rights	2	4.0%	1	2.0%
Revenues	3	6.0%	0	0.0%
Mutual insurances	4	8.0%	2	4.0%
Transports	3	6.0%	0	0.0%
Sexual life				
Libido	4	8.0%	0	0.0%
Body image	3	6.0%	0	0.0%
Shortness of breath	4	8.0%	0	0.0%
Contraception	1	2.0%	0	0.0%
Comfortable positions	0	0.0%	0	0.0%
Venereal disease	0	0.0%	0	0.0%
Pain	1	2.0%	0	0.0%
Psychological feeling				
Mood	6	12.0%	1	2.0%
Self-worth	2	4.0%	1	2.0%
Self-efficacy	2	4.0%	0	0.0%
Motivation	3	8.0%	1	2.0%
Anxiety	2	4.0%	0	0.0%
Lose interest	3	6.0%	1	2.0%
Symptoms related to the specific disease				
Pains	2	4.0%	0	0.0%
Fatigue	10	20.0%	4	8.0%
Sleep quality	2	4.0%	1	2.0%
Bladder weakness	1	2.0%	0	0.0%
Digestive gases	1	2.0%	0	0.0%
Smelly stool odour	1	2.0%	0	0.0%
constipation/diarrhea	4	8.0%	1	2.0%
Cough	6	12.0%	0	0.0%
Sputum	3	6.0%	0	0.0%
Mycosis	1	2.0%	1	2.0%
Daily life organisation				
Daily shopping/housework	3	6.0%	0	0.0%
Rest time	1	2.0%	1	2.0%
Planning of occupations/nursing	1	2.0%	0	0.0%
Hygiene	2	4.0%	0	0.0%
Self-sufficiency	3	6.0%	1	2.0%
Eating times	2	4.0%	0	0.0%
Leisure				
Leisure activity	5	10.0%	1	2.0%
Sports	8	16.0%	1	2.0%
Costs	2	4.0%	0	0.0%
Holidays	3	6.0%	0	0.0%
Vacation abroad	4	8.0%	1	2.0%
Treatments				
Regularity	4	8.0%	2	4.0%
Interruption	1	2.0%	0	0.0%
Limitation	1	2.0%	0	0.0%
Logistical supports/supply	1	2.0%	0	0.0%
Efficacy	1	2.0%	1	2.0%
Side effects	4	8.0%	1	2.0%
Disease evolution/future	8	16.0%	2	4.0%
Fertility treatment	3	6.0%	2	4.0%
Transplantation	3	6.0%	1	2.0%
Medical advances/research	6	12.0%	3	6.0%
Project				
Personal	4	8.0%	1	2.0%
Professional	5	10.0%	1	2.0%
Other	1	2.0%	0	0.0%
None domains marked	24	48.0%		
Domains considered to be unnecessary or repetitive				
Sports	1	2.0%		
Shopping/housework	1	2.0%		
Usual treatments	1	2.0%		

Data are expressed as frequency (percentage)

Patients were asked to circle up to 3 most important items among those selected. Only 17 among 50 patients (34%) selected at least one most important item. A total of 28 “most important” topics were selected, including fatigue (n = 4) and ability to conceive a child (n = 4). Domains of professional and psychological life as well as therapeutic management were also identified. Only three patients thought that some topics covered by the health care team were irrelevant and too frequently discussed (sport n = 1, shopping/housework n = 1, usual treatment n = 1). No patient wished to consider an issue other than those given in the questionnaire.

We next analysed patients’ characteristics depending on the 5 most frequently selected topics (Table 4). When compared to men, women were more frequently concerned about procreation (33% of women vs 9% of men, *p* = 0.03), study or job (40% of women vs 9% of men, *p* = 0.008) and fatigue (47% of women vs 9% of men, *p* = 0.002). In addition, patients concerned by study or job tended to be younger (22.6 ± 4.5 vs 28.5 ± 8.7 years, *p* = 0.054) and more than half suffered from diabetes (54% vs 10%, *p* = 0.022). Fatigue was selected by patients with a significantly more impaired quality of life (SGRQ total score: 37.2 ± 15.9 vs 22.3 ± 16.4, *p* = 0.014; SGRQ activity score: 45.2 ± 23.2 vs 30.3 ± 19.3, *p* = 0.048).

**Table 4:** Factors associated with main topics in CF adult patients

Factors	To have a child			Study/job			Fatigue			Sports			Disease evolution/future		
	Not selected	Selected	*P* value	Not selected	Selected	*P* value	Not selected	Selected	*P* value	Not selected	Selected	*P* value	Not selected	Selected	*P* value
Number	42	8		41	9		40	10		42	8		42	8	
Male	32	3	0.029	32	3	0.008	32	3	0.002	31	4	0.178	30	5	0.614
Age (years)	28.5 ± 8.6	22.1 ± 4.7	0.050	28.5 ± 8.7	22.6 ± 4.5	0.054	27.5 ± 8.4	28.0 ± 10.6	0.879	27.7 ± 7.8	26.0 ± 11.3	0.572	28.0 ± 8.4	25.5 ± 10.7	0.459
BMI (kg/m^2^)	21.7 ± 3.2	21.6 ± 2.8	0.854	21.5 ± 2.9	22.6 ± 3.9	0.462	22.0 ± 3.5	21.2 ± 2.6	0.532	21.7 ± 2.8	21.7 ± 4.7	0.900	22.0 ± 3.3	20.7 ± 3.1	0.293
Student/working	31	5	0.442	30	6	0.609	30	6	0.280	30	6	0.915	30	6	0.915
Pancreatic insufficiency	33	7	0.563	32	8	0.462	31	9	0.377	32	8	0.123	33	7	0.563
Diabetes	15	2	0.558	11	6	0.022	13	4	0.654	13	4	0.297	15	2	0.558
Depression	6	2	0.449	6	2	0.574	7	1	0.563	8	0	0.178	8	0	0.178
SGRQ															
Total	24.9 ± 17.1	30.2 ± 20.1	0.410	25.4 ± 18.4	27.7 ± 14.6	0.677	22.3 ± 16.4	37.2 ± 15.9	0.014	24.8 ± 17.4	30.8 ± 18.5	0.351	25.4 ± 16.8	26.0 ± 20.6	0.931
Impact	18.2 ± 16.2	28.8 ± 12.3	0.128	20.5 ± 17.6	17.3 ± 7.7	0.643	17.0 ± 15.5	27.7 ± 14.7	0.065	19.1 ± 16.5	22.9 ± 14.7	0.529	18.3 ± 15.7	29.6 ± 14.1	0.138
Activity	32.6 ± 20.0	41.5 ± 28.1	0.297	32.0 ± 20.8	41.5 ± 23.3	0.222	30.3 ± 19.3	45.2 ± 23.2	0.048	32.1 ± 20.8	43.7 ± 23.3	0.175	32.1 ± 21.2	44.0 ± 18.0	0.202
Symptoms	45.6 ± 20.0	45.6 ± 26.1	0.934	45.7 ± 20.5	45.3 ± 24.3	0.980	43.0 ± 20.6	52.2 ± 19.9	0.218	45.4 ± 20.5	47.1 ± 24.8	0.786	46.9 ± 19.7	36.5 ± 24.1	0.199
Exacerbation in last year	2.5 ± 1.4	2.0 ± 1.0	0.529	2.4 ± 1.4	2.0 ± 1.0	0.608	2.4 ± 1.5	1.9 ± 0.9	0.282	2.4 ± 1.4	2.4 ± 0.9	0.854	2.2 ± 1.5	2.6 ± 0.5	0.594
FEV_1_ (%)	61 ± 31	77 ± 25	0.202	61 ± 30	75 ± 29	0.239	64 ± 30	63 ± 33	0.898	65 ± 31	58 ± 27	0.544	66 ± 30	57 ± 27	0.447
Colonization by PA	17	3	0.835	18	2	0.209	15	5	0.508	17	3	0.835	16	4	0.563

Data are expressed as frequency or mean ± standard deviation

BMI: body mass index; FEV_1_: Forced expiratory volume at first second; PA: *Pseudomonas aeruginosa*; SGRQ: St George’s Respiratory Questionnaire

## Discussion

Using an individual simple questionnaire, we identified a number of various topics designated by patients to be discussed with health care team. Even if some topics of this questionnaire such as cough or fatigue are frequently assessed in CF, many topics appeared different from the topics addressed during regular visits usually focusing on life threatening complications like chronic respiratory failure and malnutrition.

More than half of the patients indicated at least one topic with 10% selecting more than 10 topics, highlighting frequent uncovered needs during regular follow-up visits. A few previous studies investigated CF specific needs and association with clinical characteristics [6–8, 13, 14]. Those studies used questionnaires filled during a follow-up visit or sent by mail and mainly focused on medical topics [6, 7]. Despite differences in methods, our study confirmed some previously identified unmet needs: Sawicki et al. [6] described the most frequent unmet needs as “decreased energy” (32%), “new CF therapies” (31%), “unpredictability of the future” (28%), “CF worsening” (27%) and “medication side effects” (27%). Fatigue is a very frequent symptom in CF, considered as moderate to severe in 56% of cases [8]. Its associations with clinical and functional patients’ characteristics is controversial but its impact on health status has been showed [15, 16]. In our study, we identified a clear association between fatigue as an unmet need and quality of life impairment, highlighting the importance fatigue screening.

Information about procreation was frequently selected, especially by female and youngest patients. Previous survey identified information about procreation as a need for CF patients [6, 7]. Patients’ need for discussion about procreation should be periodically assessed during visits and not restricted to urgent need expressed by patients facing this issue. Another major unmet topic to discuss raised by this study is the difficulties encountered in studies or professional activities. Patients’ employment status in our study was similar to the Cystic Fibrosis Foundation Patient Registry with 70% of student or working patients [3]. In our study, professional or scholar worries were not associated with employment status. CF can have important impact on professional life. Previous studies reported that 40% of CF patients quit their job because of the disease, 47% declared that CF affected their career choice, 24% changed duties and 23% suffered from workplace discrimination [17]. Surprisingly, in a cohort of 73 patients diagnosed with CF after 18 years, the item of employment/insurance was considered as of moderate interest [13]. In our study, more than half of the patients who were concerned about study or job suffered from diabetes. Quality of life has been shown to be significantly impaired in CF-related diabetes, especially when requiring insulin [18]. Our results suggest that CF-related diabetes may also be associated with more concerns regarding study or job. To our knowledge, the impact of CF-related diabetes on professional activity or studies has not been previously evaluated, justifying additional studies in this field.

Our study highlighted very different needs among CF adult population. Half of patients expressed no specific needs. Those patients were not different regarding demographical, clinical and functional characteristics. Lack of responses may reflect either a lack of interest (however, no patient declined the study) or absence of unmet management needs. It should also be pointed out that identified unmet needs are very disparate. Indeed, sixty items among the 62 proposed have been selected at least once. In the study of Obregon et al. [14], none of the main unmet supportive care needs (anxiety, sadness, pain and worries about the future) were associated with pulmonary impairment, BMI and pulmonary exacerbation in prior year but with age, income and or religion. Given the absence of associations between CF phenotype and patient needs, a systematic use of an educational questionnaire during follow-up visits could help to identify unmet needs.

Some topics were rarely selected in our study. Patients considered themselves as well-informed regarding treatment (interruption, limitation, logistical supports/supply, efficacy) and daily life organization. CF patients usually report a high level of confidence in their abilities in nutritional care and role of enzymes in CF therapy [7]. Sawicki et al. [6] reported that the needs in information regarding advance care planning, lung transplant, genetics, and nutrition were low. We can speculate that needs may have changed over time. Thirty years ago, most of patients reported that problems related to daily life with CF were not discussed [19]. Patients may be reluctant to approach concerns related to sexual life. None of the previous studies about expectations of CF patients analyzed this topic [4, 6–8, 13]. In a recent study, only one-third of interprofessional CF providers (physicians, nurses, social workers and other disciplines) reported being comfortable with sexual and reproductive health for adolescent and young adult women [20]. A majority of women confirmed never receiving or discussing sexual and reproductive health care with their CF team [21].

There are limitations to our study. First, our sample size is relatively small which could limit the generalizability of our results. However, clinical and functional characteristics of our cohort were similar to other studies regarding BMI [22], prevalence of exocrine pancreatic insufficiency [23], diabetes [24], *Pseudomonas aeruginosa* infections [12] and FEV_1_ [25]. Second, the questionnaire developed by a therapeutic education group has not been evaluated yet, and our study has to be considered as a pilot exploratory study. Of note previous studies have also used or modified some questionnaires not validated in CF and developed in other diseases such as cancer [4, 8, 14]. Finally, we have not assessed if the specific needs identified by this questionnaire led to a specific management. It would be interesting to analyse the impact of this custom questionnaire on therapeutic management of CF patients in a larger cohort.

## Conclusions

This study highlights that needs of CF adult patients extend beyond respiratory, nutritional concerns and treatment adherence. Despite frequent follow-up visits, half patients are still awaiting to discuss many and various issues. They express in particular an on-going interest for medical problem such as fatigue, procreation and disease evolution but also for social and professional issues. Even if two third of patients have an employment status, our study shows a high level of worry about future. Health care team should periodically individually identify CF patients’ needs and not neglect any issues. An individual educational questionnaire may help the health care team to identify specific needs in CF adults. However, the interest of this questionnaire by health care team for improving therapeutic management of CF patients remains to be validated.

## Data Availability

The datasets used and/or analysed during the current study are available from the corresponding author on reasonable request.

## Abbreviations

BMI: Body mass index
CF: Cystic fibrosis
FEV_1_: Forced expiratory volume at first second
PA: *Pseudomonas aeruginosa*
SGRQ: St George’s Respiratory Questionnaire
